# Markers of cellular senescence. Telomere shortening as a marker of cellular senescence

**DOI:** 10.18632/aging.100871

**Published:** 2016-01-23

**Authors:** Alexandra Bernadotte, Victor M. Mikhelson, Irina M. Spivak

**Affiliations:** ^1^ Karolinska Institute, Department of Medical Biochemistry and Biophysics, Stockholm, 14157, Sweden; ^2^ St. Petersburg Institute of Bioregulation and Gerontology, Russian Academy of Sciences, Saint-Petersburg, 197110 Russia; ^3^ Institute of Cytology, Russian Academy of Sciences, Saint-Petersburg, 194064, Russia; ^4^ Saint-Petersburg's State University, Saint-Petersburg, 199034, Russia; ^5^ Saint-Petersburg's Polytechnic State University, Saint-Petersburg, 195251 Russia

**Keywords:** cellular senescence, telomere, replicative senescence, aging, biomarkers, Telomere theory

## Abstract

The cellular senescence definition comes to the fact of cells irreversible proliferation disability. Besides the cell cycle arrest, senescent cells go through some morphological, biochemical, and functional changes which are the signs of cellular senescence. The senescent cells (including replicative senescence and stress-induced premature senescence) of all the tissues look alike. They are metabolically active and possess the set of characteristics *in vitro* and *in vivo*, which are known as biomarkers of aging and cellular senescence. Among biomarkers of cellular senescence telomere shortening is a rather elegant frequently used biomarker. Validity of telomere shortening as a marker for cellular senescence is based on theoretical and experimental data.

## INTRODUCTION

Cellular senescence describes how cells can induce an irreversible proliferation disability, which is resistant to growth or proliferation factors [1]. While there is no consensus on the process of aging and associated mechanisms, the scientific community has brought an idea of a senescence cellular phenotype, which is characterized by specific changes that non-immortal cells undergo during the aging. Aside from cell cycle arrest, senescent cells undergo morphological, biochemical, and functional changes, which are signs of cellular senescence.

Senescent cells of all tissue types (including those undergoing replicative and stress-induced premature senescence) look alike and show similar characteristics. Specifically, senescent cells are metabolically active and possess the set of characteristics *in vitro* and *in vivo*, which are known as biomarkers of cellular senescence. Some of the markers have strong connections with the aging theories.

However, telomere shortening, a rather elegant biomarker, is frequently used to indicate cellular senescence. Telomeres are complexes composed of proteins and nucleotides of TTAGGG repeats at the ends of eukaryotic chromosomes. Due to the mechanism of replication, telomeres shorten with every cell division, ticking as a cellular “molecular clock” [2-8].

In studying the primary mechanisms of aging, telomeres are considered by most scientists to be indicative of aging in addition to being a potential factor in determining life expectancy. Telomere theory, a major aging theory, is based on this telomeres shortening mechanism [6, 7, 9] and indicates telomeres as a promising marker of cellular senescence. Perhaps the best marker of aging is actually the cause of aging.

### Telomere theory

Telomere theory depends on three specific principles, the three pillars the Telomere theory stands on: first, that aging is programmed; the second being that irreversible cell cycle arrest happens in response to the telomere shortening; and lastly, that the total number of cell divisions in the absence of telomerase activity cannot exceed a particular limit termed the Hayflick limit [6, 7, 10, 11].

### Telomere structure and Mechanism of telomere shortening

Structurally, telomere complexes with highly repetitive clusters 5(0002032)-TTAGGG-3(0002032) (for vertebrates) form tails at the end of the chromosome. This base sequence is universal and consistent among most species. However, telomere length is species-specific and varies between 4000 and 15000 nucleotides. At the 3′ end of telomeres, there is no antiparallel strand. The single strand tail length varies between approximately 100 and 280 nucleotides in humans [12-17]. This single strand forms a telomere hairpin loop structure, termed a T-loop, and is often accompanied by numerous proteins, which together works to cap DNA and aid in preventing fusion or damage of the chromosome ends [18-20]. Moreover, this capping structure can form a G-quadruplex structure from two or four parallel or antiparallel single strands [21].

With each replication, telomeres are shortened due to the inability of DNA-polymerase to work on single-stranded 3′ ends. This mechanism of replication and associated telomere shortening was initially proposed by Olovnikiov in 1971 and was later confirmed experimentally by Blackburn [6, 7, 10].

As soon as this process of under-replication reoccurs during each of the divisions, despite telomerase-dependent and independent mechanisms of telomere elongation, telomere shortening is a replication “timer”. Critical telomere shortening (12.8 repeats of repetitive clusters) either as a loss of telomere-capping proteins results to telomere dysfunction with consequent chromosomes fusion and genome instability [22]. However, when telomere reached critical point of shortening it leads to cell cycle arrest, cellular senescence, and apoptosis – conditions, which can be considered as protection from to telomere dysfunction consequences [23, 24]. Nevertheless, cells that bypass cell cycle arrest due to oncogene activation and/or telomerase activation can undergo malignization [18, 21, 25-27].

Cell cycle arrest can occur even with lengthy telomeres. It is likely that telomere utilization speed and intensity of telomere loss also affects cell cycle arrest but not just a degree of telomere shortening. Telomeres can also be shortened as a result of DNA double-strand break damage. The cellular response mechanism to telomere shortening is similar to that common cellular response to DNA double- strand breaks; both mechanisms go through the ATR-dependent H2AX phosphorylation [28]. Though, the cell cycle arrest occurs in the same manner whether a DNA double-stranded break occurs or telomere shortening occurs. Thus, the cell cycle arrest is a universal process and starts each time there is a risk of malignization or comes to the final point of cell development – aging. This unites the majority of aging theories with mutual pathogenesis.

### Validity of telomere shortening as a marker for cellular senescence

#### 1. Telomere theory basis

The best marker of cellular senescence should provide insight into the root causes of aging. Telomere theory considers telomere shortening to be the main trigger of aging. Another prominent theory is the theory of oxidative stress (or mitochondrial theory), a major competitor of the telomere theory. Adepts of either theory realize the significance of telomere shortening as unshakable evidence in support of telomere theory. And the whole fight between tiger and lion is wither the telomere shortening is the best marker of cellular senescence, and thus, the telomere theory explains aging in a superior manner.

One strong argument against the telomere theory is based on the differences in telomere length in various tissues of the same organism and the immense variance among organisms of the same age. Indeed, telomere length, like many other continuous parameters, may not indicate specific information when considered on its own, but it may provide insight if analyzed in combination with other factors. Indeed, the length of the telomere “akin” to the human height, which as if used as only one parameter can barely tell us anything about the age of the occasion person, when it can tell us a lot if we following this person along the life.

#### 2. Telomere length reflects proliferative activity changing with age

Telomere length reflects cells' proliferative ability, which changes over time. It was shown that telomeres in highly proliferative somatic tissues, such as: human fibroblasts, leukocytes, and adipocytes were shorter than in cells of non- or low rate proliferative tissues [29].

Despite telomerase activity, the same trend is observed in stem cells. As cells proliferate during ontogenesis at varying rates serving different needs, it is reasonable to expect that telomere shortening occurs at a faster rate during earlier ages and slows down in mature ages. Indeed, hematopoietic stem cells were shown to replicate approximately 42 times per year in newborns with a telomere loss of 1.2 kb until reaching the age of three and 0.7 times and 0.5 kb per year respectively until age 13 [30].

As mentioned previously, initial telomere length can vary between individuals, while the rate of telomere shortening reflects a replicative exhaustion during aging. In mammalians telomeres are shortened at the same rate in all tissues of the same particular organism; an individual with initially longer telomeres has an upon average telomeres length across somatic tissues compared with organisms at the same age [29, 31].

#### 3. Age-related shortening

Literature addressing a relationship between telomere length and associated risk of age-related pathology and life expectancy are inconsistent. Some studies did not find a significant correlation between average telomere length and mortality risk in the elderly [32, 33]. Conversely, many studies have shown a dependence of telomere length on age: average telomere lengths are shorter in senescent cells than in cells of younger age groups and are also shorter for male than for female [12-17].

Nevertheless, telomere lengths between individuals of a particular age can vary greatly. It has been suggested that telomere length is an individual characteristic [34]. Additionally, telomere length can be dynamically change throughout an individual's life period in response to environmental factors and stress [35-37].

While this controversial data seems contradictory to telomere theory, the data is not as dramatic or surprising when considering patterns at the tissue level. It is reasonable to consider the amount of cells with crucially shortened telomeres in certain tissues or organs as a whole system instead of merely considering average telomere length overall; cells in the tissue do not exhaust their proliferative potential in a simultaneous manner. While some cells divide, others are in non-dividing conditions with a dormant ability to support the proliferative potential of the tissue. When one cell exhausts its proliferative ability, other cells can then be recruited. The more cells undergo irreversible cell cycle arrest, the less proliferative potential of the tissue remains.

Moreover, the shortest telomere (and not average telomere length) among those 256 telomeres (4 telomeres on each chromosome), turns cell to the senescence [38]. The mechanism behind replicative arrest on telomere shortening is similar to the DNA-damage signaling pathway where the size of the DNA defect is more devastating than anything else. Thus, in order to consider telomere potential of the tissue, it is necessary to determine and evaluate the amount of the shortest telomeres instead of average telomere length.

Certainly, proliferative potential and activity depend on tissue function and intensity of telomere loss. Stressful conditions forcing organs or tissues to waste proliferative potential can lead to earlier aging onset, which reflects telomere length [33].

It is obvious that it is not necessary for all cells of a certain tissue to undergo irreversible cycle arrest at once. After a sufficient amount of cells undergoes senescent conditions, tissues cannot function at the same level and the vicious cycle of aging begins. After that cells and tissues cannot properly perform functions in a stressful extracellular environment that consumes the tissues' proliferative potential.

Nevertheless, a strong correlation was found between mean telomere length in early life and lifespan [16], and even between mean telomere length and paternal lifespan if inherited by female offspring in humans [39].

#### 4. Age-related diseases

Telomere shortening and an associated depletion of cells' proliferative potential may be sufficient to cause the onset of many diseases associated with aging [40, 41]. Studies investigating telomere biology have demonstrated that telomeres and telomere-associated proteins play an important role in the aging process and that accelerated telomere erosion is associated with metabolic and inflammatory diseases associated with aging [42] Many neurodegenerative disorders have been shown to feature early cell death. As telo-mere shortening is related to premature cellular senescence, telomere length may be an effective marker of cellular pathology in neurological diseases; specifically, it has been demonstrated in dementia, Huntington's disease, and ataxia telangiectasia. Of these studies, the shortest telomere lengths were associated with Huntington's disease [43]. Additionally, an inverse correlation with the length of telomeres was shown for the development of cardiovascular disease [44-47], renal failure [48, 49], various cancers [50, 51], Alzheimer's disease [52], and Parkinson's disease [53]. In most cases, a direct link between telomere shortening and a constant high level of oxidative stress was observed in subjects with these diseases [54].

Interestingly, some studies contain data on long-term observations of initially healthy men and include time of disease onset. One research group monitoring individuals' telomere length over a period of 15 years showed what proportion of respondents developed type 2 diabetes [55] or cancer [54, 56]. Both diseases were shown to be significantly more likely to occur in patients with shorter initial telomere lengths. It has also been shown in longitudinal studies that telomere shortening is frequently accompanied by a decrease in muscular strength [57] and weight loss. Reduced telomere length may be indicative of biological pathways shared between disorders contributing to cellular senescence. It is most likely that this shortening is associated not only with accelerated replicative aging, but also with the failure of telomere repair processes [58].

#### 5. Short telomere syndromes

Such studies of an extended range of diseases, telomere length, and premature aging act as conspicuous conformation of the validity of telomere length as a marker of cellular senescence. Progerias or premature aging, or the short telomere syndromes include genetic diseases associated with short telomeres and premature senescence [59]. Specifically, these diseases include: Hutchinson-Gilford syndrome, Dyskeratosis congenita (also referred to as Zinsser-Cole-Engman syndrome), ataxia-telangiectasia (or Louis–Bar syndrome), Hoyeraal–Hreidarsson, and Revesz syndrome. These syndromes are all characterized by dramatically shortened telomeres as well as premature replicative senescence with a decreased Hayflick limit in cell cultures of patients and aging phenotypes, which develops much earlier in such patients than in the healthy individuals [60-62]. There is a clear correlation between affected telomere length and aging in progerias.

Thus, whether these syndromes are applicable as a model of aging is a relevant inquisition. Most researchers define aging as an age-progressive persistent decline in physiological functioning with an associated decrease in survival rate (increased mortality risk in demographic aging) and reproductive ability. Indeed, all previously mentioned syndromes are characterized by an age-progressive persistent decline in organ and tissue function, reproductive function, increased mortality risk, and predisposition to malignancies – all of which are phenotypic symptoms of human aging [63]. Furthermore, telomere shortening observed in these syndromes is not only a symptom of the disease, but also the cause.

#### 6. Association with other markers of cellular senescence

All markers of cellular senescence, including telomere shortening, have limitations; while some markers may be informative in certain conditions, it may not always be the case in other circumstances. The best marker, however, should address both *in vivo* and *in vitro* conditions, apply to various tissues and species, and show consistency and overlapping with other reliable markers. Telomere shortening meets these requirements and shares similar trends with a majority of various other senescence-associated cellular markers.

##### 6.1. Senescence-associated β –galactosidase and telomere shortening

Senescence-associated increased activity and expression of the lysosomal hydrolase β-galactosidase remains the most popular marker of cellular senescence, particularly of replicative senescence, due to its usefulness [64-66]. Enzyme activity of β-galactosidase in normal (young) cells can be detected using 5-bromo-4-chloro-3-indolyl β-D-galacto-pyranoside (X-Gal) at pH 4; activity of the enzyme (or its isoform) in senescent cells, however, is observed at pH 6 and is termed senescence-associated β-galactosidase (SA-βgal) [64, 67, 68]. Enhanced activity and expression of β–galactosidase as aging continues was shown in vitro for multiple species and cell types [64, 67-70]. Nevertheless, the universality or reliability of senescence-associated β–galactosidase as a marker of senescence remains. Studies of cells with defective lysosomal β-galactosidase have shown that β–galactosidase does not trigger cellular senescence [71, 72] and only accompanies senescent alterations. However, input of β-galactosidase seems to correlate with telomere alterations. The number of β-galactosidase stained cells was found as a function of population doubling level (PDL) on human retinal pigment epithelial cells; while simultaneously telomere length, which was observed in a stage of 12 PDL and in old culture of 57 PDL, shrank from 10 kb to 4kb respectively [73].

##### 6.2. Senescence-associated heterochromatin foci and telomere shortening

Telomere shortening correlates with senescence-associated heterochromatin foci (SAHF), which are facultative heterochromatin domains associated with irreversible cell cycle arrest and accumulate in senescent cells [74, 75]. Structurally, SAHF contain silent hetero-chromatin domains consisting of a di-or tri-methylated lysine 9 of histone H3 (H3K9Me2/3), a histone H2A variant (macroH2A), and heterochromatin protein 1 (HP1) proteins [74, 76, 77]. SAHF is not a universal marker of aging cells, but it is tissue and species dependent senescent marker [74, 78]. Formation of SAHF may be spontaneously stochastic and program-med; it can be induced in senescent cells by oncogenes, toxic agents, or telomere shortening [74, 76, 79-81].

##### 6.3. Histone γ-H2AX and telomere shortening

One SAHF is usually considered separately to indicate senescent cells due to its specificity. Histone γ-H2AX (a replacement histone) is phosphorylated at the C-terminal serine-139 of the H2AX histone. This is the second most common marker of cellular senescence after SA-βgal. Histone γ-H2AX is the most sensitive marker of double-stranded DNA breaks (DSBs) and telomere shortening [28, 82]. The number of γ-H2AX foci increases in damaged and senescent cells in most tissues and species, both *in vivo* and *in vitro* [83-85]. Correlation with telomere shortening is corroborated by accumulation of γ-H2AX foci in cells of older donors and patients with progeria [82-84, 86-88].

Indeed, γ-H2AX foci have a robust correlation with replicative telomere shortening. As with 53BP1, it forms telomere dysfunction-induced foci. When multiple foci of this type are expressed in a cell (observed on fibroblasts and epithelial cells), cellular p53-dependent replicative arrest has been induced [17]. In this specific case, this marker has a strong connection with telomere theory.

On the other hand, some studies of this marker have contributed evidence that may support oxidative theory; specifically, results suggest that DNA damage (including DSBs) accumulate during aging, according the oxidative theory. Each single DSB is marked by γ-A2SAX foci, a highly sensitive marker, during all phases of the cell cycle [82, 84, 86, 87, 89, 90].

Thus, γ-H2AX may also be an effective marker of telomere shortening. However, γ-H2AX may not be a superior marker of cellular senescence due to a lack of specificity. Experimental results have indicated that γ-H2AX foci colocalize with DSB repair factors (such as Rad51, BRCA1, Mre11, Rad50, and Nbs1) [86, 91] and participate the repair of DSBs [85, 89, 90]. Therefore, we can conclude that the amount of γ-H2AX foci can be reduced following DSB repair. Indeed, it was reported that the formation of telomere dysfunction-induced foci accompanied by γ-H2AX is reversible and the number of telomere dysfunction-induced foci can be reduced during the early G1 phase of the cell cycle [17]. Thus, γ-H2AX foci reflect – not only age-dependent irreversible DSBs (including telomere shortening) – but also temporal accumulation of DSBs, which may be a result of massive ionizing radiation (1 Gy of ionizing radiation, about 2 × 106 bp of DDBs, triggers phosphorylation of about 1% of the H2AX) [90]. Hence, γ-H2AX can be misleading in cases where there is high risk of ionizing radiation and some cases may show discordance with other senescent markers.

Histone γ-H2AX as a potential marker of cellular senescence combines two major aging theories. This marker can be accompany telomere shortening but should not be considered as a stand-alone marker of cellular senescence.

##### 6.4. Epigenetic modifications and Telomere shortening

Methylation of DNA and histones is a marker of cellular senescence and contributes to another major theory of aging: gene silencing theory [92]. Histone methylation in the telomere region and demethylation of the human telomerase catalytic subunit (hTERT) both play significant roles in maintaining heterochromatin, transcription silencing at telomeres, and telomerase inactivation [93-96]. However, one factor contributing to the value of telomere shortening as a marker is the observed correlation between DNA methylation and telomere length [97].

##### 6.5. p53-binding protein and Telomere shortening

Another traditional marker of the DNA repair processes is expression of p53-binding protein 1 (53BP1) [98]. The dynamics of 53BP1 in response to DNA damage generally coincides with those of γ-H2AX and they are frequently colocalized in DNA double-stranded break zones. Phosphorylation of 53BP1 at the serine residue at position 1219 (S1219) occurs in response to DNA damage, including telomere shortening [99]. The appearance of 53BP1 in the presence of telomere shortening is another reliable marker of senescent cells, which again vindicates validity of telomere shortening as a marker for cellular senescence.

##### 6.6. Promyelocytic leukemia nuclear bodies (PML-NBs) and Telomere shortening

An increase in the amount of promyelocytic leukemia nuclear bodies (PML-NBs) is another marker of cellular senescence [100] and can be induced during premature senescence [101]. In the absence of telomerase, telomeres can be elongated via the alternative mechanism of lengthening of telomeres, which is going strongly in the presence on PML-NBs. On the other hand, PML-NB formation at telomeres induces telomere clustering and promotes RAS-induced premature senescence [101, 102]. However, telomere shortening and PML-NBs have vast overlapping and may both be effective as markers of cellular senescence.

## Conclusion

Summarizing theoretical and experimental data we can conclude that among biomarkers of cellular senescence the telomere shortening is a cellular senescence biomarker of choice. When Telomere theory provided theoretical base of telomere marker, experimental data of age-related telomere shortening, age-related diseases, telomere shortening in progerias, and strong association with other markers of cellular senescence made the telomere shortening elegant valid, and useful biomarker of cellular senescence, which is reflecting the replicative cellular and tissue potential. However, regarding the telomere measurement methods it is reasonable to consider the amount of shortened telomeres in tissues as indicator of cellular and tissue proliferative potential instead of average of telomere length.
